# Unique mechanisms of connective tissue growth factor regulation in airway smooth muscle in asthma: Relationship with airway remodelling

**DOI:** 10.1111/jcmm.13576

**Published:** 2018-03-07

**Authors:** Junfei Wang, Alen Faiz, Qi Ge, Cornelis J. Vermeulen, Joanne Van der Velden, Kenneth J. Snibson, Rob van de Velde, Sonia Sawant, Dikaia Xenaki, Brian Oliver, Wim Timens, Nick ten Hacken, Maarten van den Berge, Alan James, John G. Elliot, Liang Dong, Janette K. Burgess, Anthony W. Ashton

**Affiliations:** ^1^ Department of Pulmonary Medicine Qilu Hospital of Shandong University Jinan Shandong China; ^2^ Woolcock Institute of Medical Research University of Sydney Sydney NSW Australia; ^3^ University of Groningen University Medical Center Groningen Department of Pulmonary Diseases Groningen The Netherlands; ^4^ University of Groningen University Medical Center Groningen GRIAC (Groningen Research Institute for Asthma and COPD) Groningen The Netherlands; ^5^ University of Groningen University Medical Center Groningen Department of Pathology & Medical Biology Groningen The Netherlands; ^6^ Discipline of Pharmacology The University of Sydney Sydney NSW Australia; ^7^ Faculty of Veterinary and Agricultural Science Melbourne Veterinary School University of Melbourne Parkville Vic. Australia; ^8^ School of Life Sciences University of Technology Sydney NSW Australia; ^9^ Department of Pulmonary Physiology and Sleep Medicine Sir Charles Gairdner Hospital Perth WA Australia; ^10^ School of Medicine and Pharmacology The University of Western Australia Perth WA Australia; ^11^ Division of Perinatal Research Kolling Institute of Medical Research Sydney NSW Australia

**Keywords:** airway remodelling, airway smooth muscle, asthma, connective tissue growth factor

## Abstract

Neovascularization, increased basal membrane thickness and increased airway smooth muscle (ASM) bulk are hallmarks of airway remodelling in asthma. In this study, we examined connective tissue growth factor (CTGF) dysregulation in human lung tissue and animal models of allergic airway disease. Immunohistochemistry revealed that ASM cells from patients with severe asthma (A) exhibited high expression of CTGF, compared to mild and non‐asthmatic (NA) tissues. This finding was replicated in a sheep model of allergic airways disease. In vitro, transforming growth factor (TGF)‐β increased CTGF expression both in NA‐ and A‐ASM cells but the expression was higher in A‐ASM at both the mRNA and protein level as assessed by PCR and Western blot. Transfection of CTGF promoter‐luciferase reporter constructs into NA‐ and A‐ASM cells indicated that no region of the CTGF promoter (−1500 to +200 bp) displayed enhanced activity in the presence of TGF‐β. However, in silico analysis of the CTGF promoter suggested that distant transcription factor binding sites may influence CTGF promoter activation by TGF‐β in ASM cells. The discord between promoter activity and mRNA expression was also explained, in part, by differential post‐transcriptional regulation in A‐ASM cells due to enhanced mRNA stability for CTGF. In patients, higher CTGF gene expression in bronchial biopsies was correlated with increased basement membrane thickness indicating that the enhanced CTGF expression in A‐ASM may contribute to airway remodelling in asthma.

## 1 INTRODUCTION

Asthma is a common, chronic respiratory disease affecting more than 300 million people worldwide.^1^ The main characteristics of asthma are airway inflammation, airway hyper‐responsiveness and airway remodelling.^2^ The structural changes in the airways, termed airway remodelling, include increased airway smooth muscle (ASM) bulk, increased basal membrane thickness and vascular expansion.^3^, ^4^ The extent of airway way remodelling correlates with several clinical features of asthma^5^, ^6^, ^7^, ^8^ and agents that normalize the remodelling response potentially improve asthma symptoms.^9^, ^10^, ^11^ Once considered a manifestation of chronic inflammation, recent studies have identified remodelling is a separate but parallel component of the asthmatic process.^12^

In asthma, the observed increase in ASM cell bulk and contractility directly mediates airway narrowing and is central to the process of airway remodelling. Increased ASM number correlates with increased reticular basement membrane (BM) thickness and eosinophilia, but not neutrophilia.^13^ Further, the secretory profiles of ASM from asthmatic patients differ significantly from those of non‐asthmatic patients suggesting that paracrine signalling from the ASM in may have as much to do with airway remodelling as their contractile state (reviewed in Ref. 14). Ultimately, the increased deposition of extracellular matrix (ECM) proteins by ASM cells in asthma is key to the airway narrowing that takes place.^12^, ^15^, ^16^, ^17^, ^18^ Often this is a response to an imbalance in the cytokines/growth factors present in their local milieu.^3^

In lung tissue, ASM cells are a potent source of connective tissue growth factor (CTGF), a member of the cysteine‐rich 61, CTGF, nephroblastoma (CCN) family of proteins.^19^ Our previous studies have shown greater CTGF expression in primary asthmatic (A)‐ASM cells than non‐asthmatic (NA)‐ASM cells after TGF‐β treatment.^20^, ^21^, ^22^ CTGF controls ECM deposition and ultimately airway biomechanics through changes to collagen deposition which increase ECM density and airway stiffness.^23^ Indeed, the increased stiffness of the matrix in which asthmatic ASM cells are embedded promotes a more proliferative and pro‐inflammatory ASM phenotype.^14^

The mechanisms underlying the differential regulation of CTGF expression in A‐ASM are not currently known. Studies in other systems have reported that CTGF induction by TGF‐β is regulated through interactions of transcription factors with promoter elements directly upstream of the promoter start site.^24^, ^25^, ^26^, ^27^ In this study, we investigated the mechanisms that enhance TGF‐β induction of CTGF release from A‐ASM cells and the potential links to airway remodelling in asthma.

## 2 MATERIALS AND METHODS

### 2.1 Primary ASM cell isolation and culture

Approval for experiments with human lung tissue was provided by the Ethics Review Committee of the South West Sydney Area Health Service, St Vincent's Hospital Sydney, Strathfield Private Hospital, Royal Prince Alfred Hospital and the University of Sydney Human Research Ethics Committee. Primary human ASM cells were obtained through dissection of donated lung tissue following transplantation and from endobronchial biopsies from volunteers who provided written informed consent, as described previously.^28^, ^29^ The patients' details are described in Table 1.

**Table 1:** Details of asthmatic and non‐asthmatic donors

Patient	Age	Sex	Diagnosis	Sample type
1	67	Male	Healthy	Transplant
2	47	Male	Healthy	Transplant
3	22	Female	Healthy	Biopsy
4	64	Male	ILD	Transplant
5	76	Male	Pulmonary cryptococcosis	Resection
6	61	Female	Pulmonary hypertension	Transplant
7	41	Female	Adenocarcinoma	Resection
8	29	Male	Healthy	Biopsy
9	16	Male	Healthy	Transplant
10	65	Male	Cancer	Resection
11	27	Female	Asthmatic	Biopsy
12	54	Male	Asthmatic	Biopsy
13	23	Male	Asthmatic	Biopsy
14	58	Male	Asthmatic	Biopsy
15	21	Male	Asthmatic	Biopsy
16	38	Male	Asthmatic	Biopsy
17	61	Female	Asthmatic	Biopsy
18	85	Male	Asthmatic	Biopsy
19	51	Male	Asthmatic	Biopsy
20	63	Male	Asthmatic	Biopsy
21	59	Female	Asthmatic	Biopsy
22	64	Male	Asthmatic	Biopsy
23	NA	NA	Healthy	Transplant
24	52	Male	Cancer	Resection
25	22	Male	Healthy	Biopsy
26	60	Female	Cancer	Resection
27	27	Male	Asthmatic	Biopsy
28	33	Male	Asthmatic	Biopsy
29	50	Male	Asthmatic	Biopsy

ILD, interstitial lung disease; NA, not available.

Cells were grown in 10% (v/v) foetal bovine serum (FBS) (JRH Biosciences, Brooklyn, VIC, Australia)/high glucose Dulbecco's modified Eagle's medium (DMEM, Sigma‐Aldrich, St. Louis, MO, USA) containing 100 units/mL of penicillin, 100 μg/mL of streptomycin, 0.25 μg/mL of amphotericin B (Thermo Fisher, Waltham, MA, USA) and 25 mmol/L HEPES (Sigma‐Aldrich). Use of primary ASM cells was restricted to passage numbers 2 and 8.

### 2.2 Detection of CTGF by immunohistochemistry

Sections from archived paraffin‐embedded lung tissue were obtained from non‐, mild and severely asthmatic patients^3^, ^4^ as well as a sheep model of allergic airways disease that had been previously described.^30^ Slides were de‐paraffinized in xylene and rehydrated through graded ethanol solutions. Peroxidase‐blocking solution (DAKO, Agilent Pathology, Foster City, CA, USA) was used to block endogenous peroxidases at 37°C for 15 minutes. Slides were washed and incubated with serum‐free protein block (DAKO) at 37°C for 30 minutes followed by incubation with rabbit anti‐CTGF primary antibody (2.5 μg/mL for human, 5 μg/mL for sheep lung tissue, ab6922; Abcam, Cambridge, UK) or rabbit IgG (same concentration as CTGF antibody, X0903, DAKO) overnight at 4°C. Primary antibodies were diluted in REAL antibody diluent (DAKO) to minimize non‐specific binding. After washing, slides were incubated with EnVision+ system HRP labelled polymer anti‐rabbit secondary antibody (K4003, DAKO) for 45 minutes at 37°C. Liquid diaminobenzidine+ (DAB) substrate chromogen system (DAKO) was added and incubated for 10 minutes at room temperature. All slides were counterstained with haematoxylin (with eosin for human sections) before dehydration through graded ethanol and mounted with dibutyl phthalate in xylene (DPX, Tingalpa, QLD, Australia) mounting medium (VWR BDH Prolabo^®^ Chemicals). Human slides were scanned using a wide‐field FL and TL microscope ZEISS Axio Scan.Z1 SlideScanner (Zeiss, Oberkochen, Germany), and the sheep slides were scanned by a NDP scanner (HAMAMATSU, Hamamatsu, Japan).

### 2.3 ASM cell stimulation

For all experiments, unless otherwise indicated, ASM cells were seeded into 6‐well plates at a concentration of 1 × 10^4^ cells/cm^2^ in 5% (v/v) FBS/DMEM and grown for 3 days before being made quiescent in 0.1% (w/v) bovine serum albumin (BSA, Sigma‐Aldrich, St. Louis, MO, USA)/DMEM for 24 hours. Cells were treated with recombinant human TGF‐β1 protein (1 ng/mL, R&D systems, Minneapolis, MN, USA) for the indicated durations. Cells were washed twice with ice‐cold phosphate‐buffered saline (PBS, Sigma‐Aldrich) and lysed in lysis buffer prior to total RNA being extracted using the ISOLATE RNA mini kit (Bioline, London, UK) according to the manufacturer's instructions, quantified with a NanoDrop 2000 Spectrophotometer (NanoDrop Technologies, Wilmington, DE, USA) and stored in −20°C for further use. Alternately, cells were scraped into protein lysis buffer (20 mmol/L Tris‐HCl, pH 7.4, 150 mmol/L NaCl, 1 mmol/L Na_2_EDTA, 1 mmol/L EGTA, 1 mmol/L NaF, 20 mmol/L Na_4_P_2_O_7_, 2 mmol/L Na_3_VO_4_, 1% (v/v) Triton X‐100, 10% (v/v) glycerol, 0.1% (w/v) SDS, 0.5% (w/v) sodium deoxycholate, 1 mmol/L phenylmethylsulphonyl fluoride and 1:100 Protease Inhibitor Cocktail Set III [Merck Millipore, Billerica, MA, USA]) and stored at −20°C until analysis.

### 2.4 Real‐time reverse transcription polymerase chain reaction

To investigate CTGF mRNA expression, real‐time PCR was conducted on NA‐ and A‐ASM cells with primers and probe specific to CTGF (Hs01026927_g1, Life Technologies, Carlsbad, CA, USA) and BioSense SensiFast™ Probe Hi‐ROX Mastermix (Bioline) using a StepOne Plus detection system (Thermo Fisher). Relative gene expression between treatments was calculated using the 2^−∆∆Ct^ method after normalization against the 18s rRNA probe (4319413E‐1011052, Life Technologies).

### 2.5 Western blot

Total cellular protein was extracted and separated on a 10% (w/v) SDS‐PAGE gel, and transferred to polyvinylidene difluoride membrane (Merck Millipore). Membranes were blocked in 5% (w/v) skim milk in Tris‐buffered saline (TBS, 20 mmol/L Tris base, 150 mmol/L NaCl, PH7.4) containing 0.05% (v/v) Tween 20 for 30 minutes at room temperature and incubated with goat polyclonal anti‐CTGF antibody (1:1000, sc‐14939, Santa‐Cruz Biotechnology, Dallas, TX, USA) or anti‐GAPDH (1:10 000, MAB‐374; Millipore) at 4°C overnight. Membranes were washed and incubated with horseradish peroxidase‐conjugated secondary antibody (1:2000 for CTGF [P0160], 1:50 000 for GAPDH [P0161], DAKO). Images were captured using a Kodak Image station 4000 mm, and band intensity was quantified with Carestream MI SE software.

### 2.6 CTGF promoter constructs

A Gluc‐on reporter plasmid containing the full‐length CTGF promoter (−1500 to +200 bp) driving expression of a secreted Gaussia luciferase was purchased from GeneCopoeia (Cat. HPRM25713‐PG04, Rockville, MD, USA). A series of 5′ deleted constructs were generated by PCR using the primers shown in Table 2. The full‐length CTGF promoter was excised using the restriction enzymes Hind III and Bgl II (New England Labs, Ipswich, MA, USA) and the generated PCR products ligated into the vector using T4 DNA ligase (Promega, Madison, WI, USA). All constructs were sequence verified at the Australian Genome Research Facility (Melbourne, Vic., Australia) prior to expansion and transfection.

**Table 2:** Primer sequence for CTGF 5′ deletion mutant constructs

Primer name	Primer sequence (5′‐3′)
−100 forward	ATC GAG ATC TAA CAA CAT AGA TTC CAA ATG A
−400 forward	ATC GAG ATC TGT AAT GGA ATC AGA CTT CTT A
−700 forward	ATC GAG ATC TAA AAC TAA GCA AGA GTT TTG G
−1000 forward	ATC GAG ATC TCT TCA GCT ACC TAC TTC CTA A
−1300 forward	ATC GAG ATC TAT GCG AGG AAT GTC CCT GTT T
Reverse primer	ATC CGA GCT CGG TAC CAA GCT T

CTGF, connective tissue growth factor.

### 2.7 Transfection and luciferase assay

Airway smooth muscle cells and NIH‐3T3 mouse embryonic fibroblasts were seeded in 12‐well plates at densities of 1 × 10^4^ cells/cm^2^ and 8 × 10^4^ cells per well, respectively, for 24 hours in 10% (v/v) FBS/DMEM. The full‐length CTGF promoter, or the 5′ deleted constructs, were transfected into the indicated cells using Lipofectamine 3000 (L‐3000075; Invitrogen, Carlsbad, CA, USA) transfection reagent according to the manufacturer's instructions using either 1 or 1.6 μg of plasmid DNA for ASM and NIH‐3T3 cells, respectively. Cells were stimulated with TGF‐β1 (1 ng/mL for ASM and 10 ng/mL for NIH‐3T3) 24 hours after transfection, for a further 24 hours. The supernatant was collected for luciferase detection by Secrete‐Pair™ Dual Luminescence Assay Kit (GeneCopoeia) and total RNA isolated from the cell monolayers using the ISOLATE RNA mini kit (Bioline) for CTGF mRNA detection.

### 2.8 Analysis of distant transcription factor binding sites with potential to regulate CTGF promoter

To investigate other potential transcription factor binding sites in close proximity to the CTGF promotor, H3K27Ac binding (a marker of transcription factor binding) was investigated. This analysis was conducted using chromatin immunoprecipitation sequence (ChIP‐Seq) data on human umbilical vein endothelial cells (HUVECs), mouse embryonic (NIH‐3T3) and normal human lung (NHLF) fibroblasts generated as part of the Encyclopedia of DNA Elements (ENCODE) Project (GSE29611).

### 2.9 Assessment of mRNA stability in ASM cells

To measure CTGF mRNA stability, ASM cells rendered quiescent by incubation in 0.1% (w/v) BSA/DMEM for 24 hours were treated with TGF‐β1 (1 ng/mL) for 8 hours. After washing with PBS, actinomycin D (10 mg/mL, Sigma‐Aldrich) was added to the media for a further 0‐16 hours as indicated. Total RNA was isolated, and CTGF mRNA was quantified by PCR as described above.

### 2.10 CTGF gene expression relationship with clinical factors

We obtained high‐quality RNAseq data from 184 biopsies. Biopsies were derived from 77 healthy individuals and 107 current or former asthma patients. All patients originated from cohorts investigated earlier by our research group, and a set of previously acquired clinical data is available^31^, ^32^. The study protocol was approved by the University Medical Center Groningen medical ethics committee. All patients gave their written informed consent. For full patient information and details relating to RNA isolation and sequencing, refer to Appendix S1.

A linear model was fitted to CTGF gene expression derived from RNA sequencing in bronchial biopsies (expressed as fragments per kilobase of transcript per million mapped reads, FPKM) as a function of forced expiratory volume in 1 second percentage predicted (FEV1% predicted), BM thickness, log_2_(PC_20_ to methacholine) and log_2_(% of eosinophils) in sputum of asthma patients (n = 69). Age, gender and smoking status were used as correction factors. All analyses were conducted using R (version 3.3.2).

### 2.11 Statistical analysis

Data were tested for normal distribution and analysed via a 2‐way analysis of variance (ANOVA) with Bonferroni post‐test. A *P*‐value less than .05 was considered to be statistically significant (*P* < .05).

## 3 RESULTS

### 3.1 Increased CTGF expression in asthmatic lung tissue in vivo in a sheep model and exaggerated release of CTGF from asthmatic ASM cells in vitro

**Figure 1:**
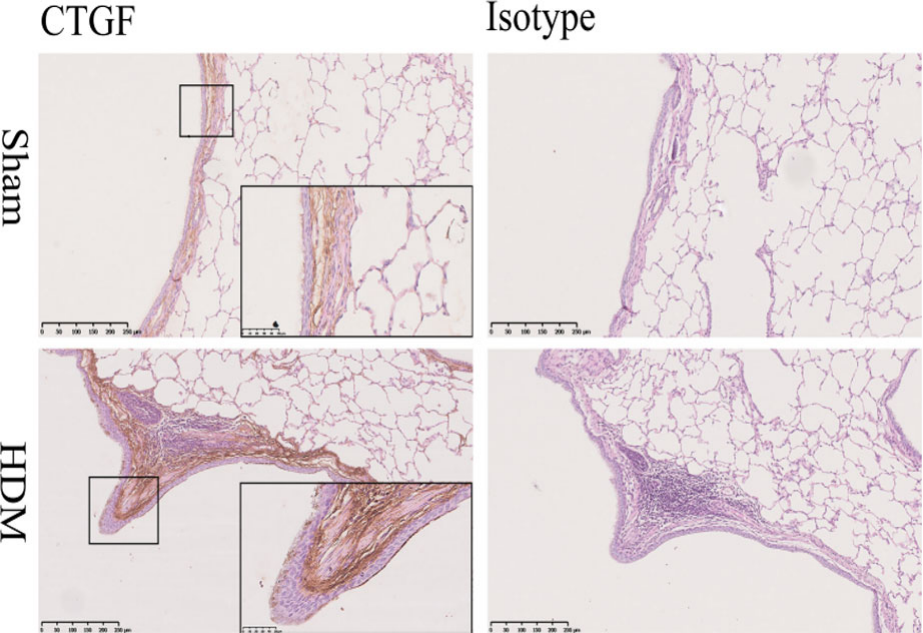
Connective tissue growth factor (CTGF) expression is increased in house dust mite (HDM)‐induced allergic airway disease in sheep lungs. CTGF expression in a model of allergic airway disease was assessed by immunohistochemistry in HDM‐ and saline‐exposed (sham control) lung segments from the same sheep^30^ (n = 5). Isotype‐matched negative control antibody on serial sections shown for comparison. Representative images shown for each group

**Figure 2:**
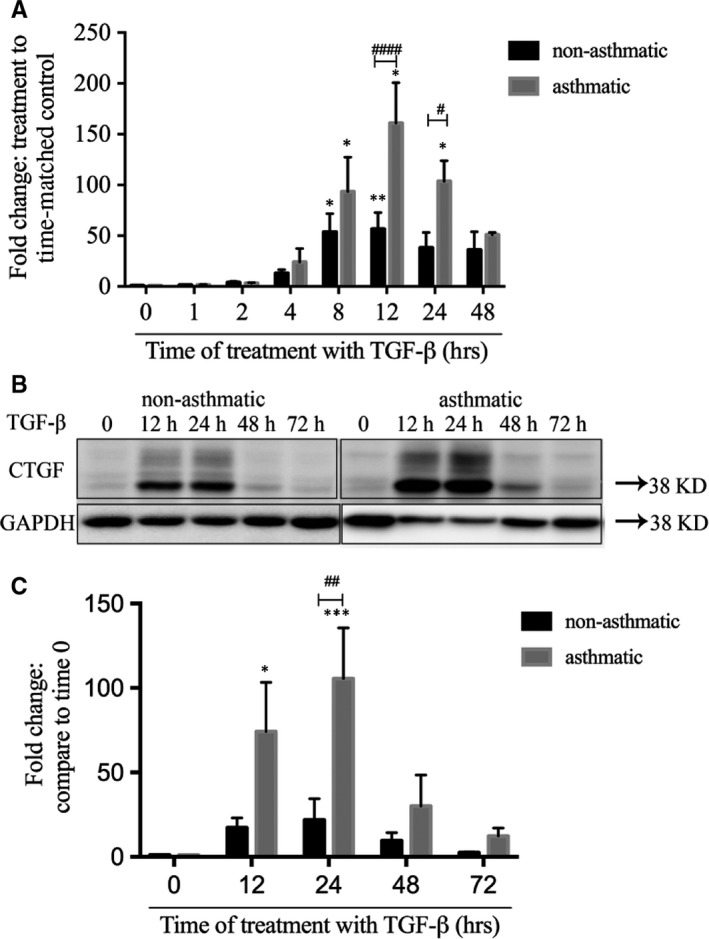
Asthmatic airway smooth muscle (ASM) cells have different kinetics of connective tissue growth factor (CTGF) induction. ASM cells from A‐ and NA‐donors were stimulated with transforming growth factor (TGF)‐β (1 ng/mL) for up to 72 h and CTGF transcript (A; NA‐ASM [n = 7] and A‐ASM [n = 5]) and protein (B; NA‐ASM [n = 4] and A‐ASM [n = 7]) levels examined by Q‐PCR and Western blot, respectively. Representative images of Western blots are shown. Changes in CTGF expression by Western blot were quantified using image J software (C). *P < .05, **P < .01 and ***P < .001 denotes significance between bovine serum albumin and TGF‐β. #*P* < .05, ##*P* < .01 and ####*P* < .0001, # indicates significant difference between NA‐ and A‐ASM cells

Immunohistochemistry showed that CTGF was detected in both sham and house dust mite (HDM)‐sensitized (n = 6 for both) sheep lung tissues, and the staining was concentrated in the ASM layer (Figure 1). Having established that asthmatic ASM shows dysregulation of CTGF expression, we sought to understand the mechanism responsible. Treatment of both asthmatic (A‐) (n = 5) and non‐asthmatic (NA‐) (n = 7) ASM cells with TGF‐β1 induced CTGF expression (Figure 2A), confirming our previous observations.^20^, ^22^ As before, the increase of CTGF mRNA in A‐ASM cells was 3‐ and 2.5‐fold greater than in NA‐ASM cells at 12 (*P* < .0001) and 24 hours (*P* < .05) after TGF‐β1 treatment, respectively. Similar kinetics were observed in CTGF protein expression (Figure 2B and C); however, the magnitude of induction was greater (5‐ and 4‐fold at 12 and 24 hours, respectively) in A‐ (n = 7) than NA‐ (n = 4) ASM cells (*P* < .01).

### 3.2 Transcriptional regulation of CTGF promoter (−1500 to +200 bp) is the same in asthmatic and non‐asthmatic ASM cells

**Figure 3:**
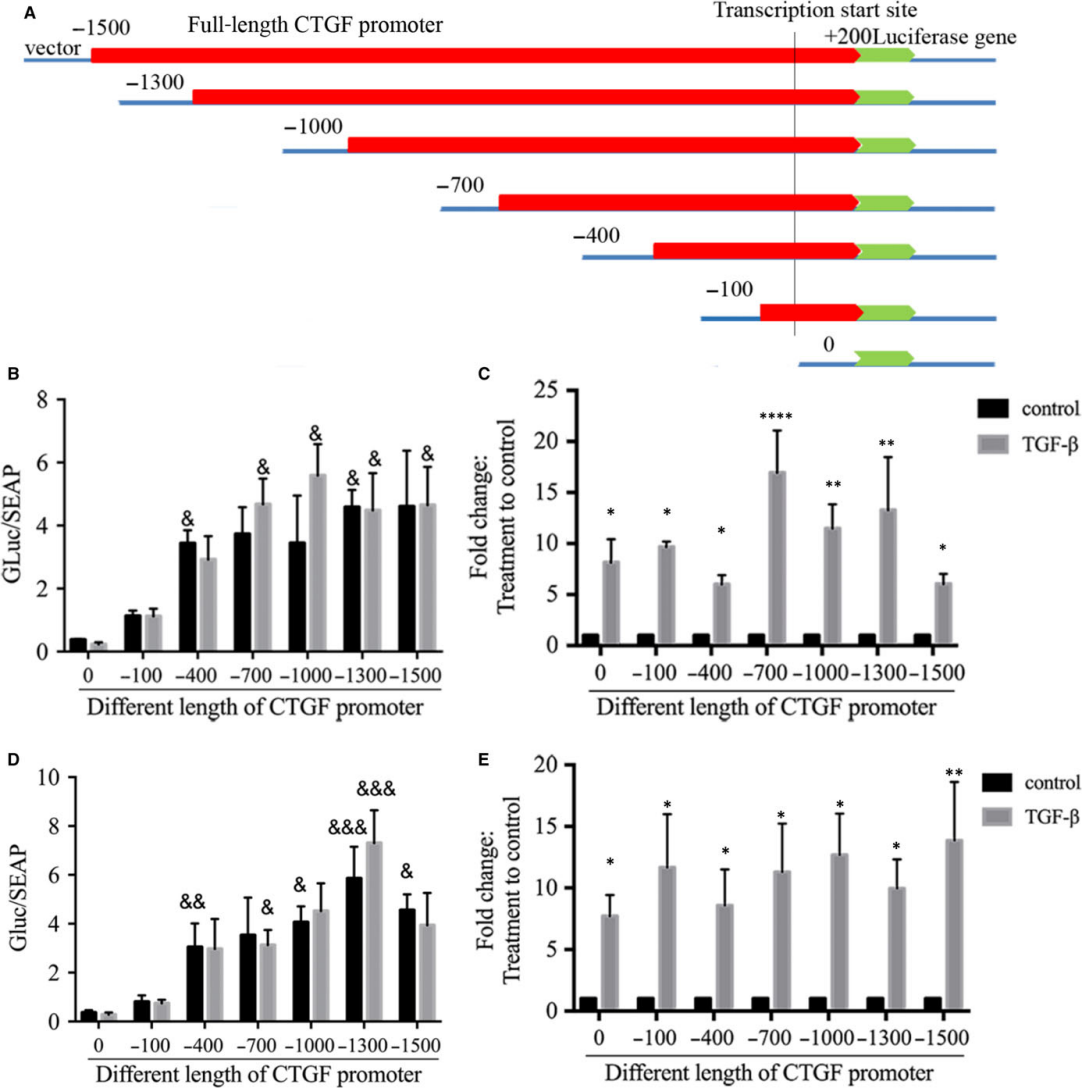
Basal regulation of the connective tissue growth factor (CTGF) promoter is the same in NA‐ and A‐ASM cells. A. Schematic of the 5′ deleted CTGF promoter constructs used to examine regulation in NA‐ and A‐ASM cells. Different lengths of the human CTGF promoter (−1500 to +200; 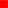) were placed upstream of a Luciferase reporter construct (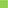). Secretion of alkaline phosphatase (SEAP) expression was driven by CMV promoter in the same construct and was used as a control for transfection efficiency. Luciferase activity in conditioned media was detected after stimulation of transfected ASM ± TGF‐β (1 ng/mL; NA‐ [B, n = 5] and A‐ASM [D, n = 5]). CTGF mRNA in NA‐ (C) and A‐ASM (E) was measured by Q‐PCR in the same cells used for luciferase assays to determine the effectiveness of induction for the endogenous gene. “&” Denotes significance between promoter‐less (0) and luciferase reporter (&*P* < .05, &&*P* < .01, &&&*P* < .001). *Indicates significant difference of CTGF mRNA expression between TGF‐β and BSA, *P < .05, **P < .01, ****P < .0001. ASM, asthmatic airway smooth muscle; BSA; bovine serum albumin

Other studies have reported that CTGF expression induced by TGF‐β is regulated by an interaction between transcription factors and CTGF promoter binding sites immediately upstream of the promoter start site. To investigate mechanisms underlying the greater CTGF release from A‐ASM cells, compared to NA‐ASM cells, after TGF‐β treatment, we transfected a full‐length (−1500 to + 200 bp), and a series of 5′ truncated, CTGF promoter‐luciferase reporter constructs (Figure 3A) into both A‐ and NA‐ASM cells. The secretion of alkaline phosphatase, driven by a CMV promoter within the constructs, was used to normalize for transfection efficiency. Basal promoter activity was observed with the (−400 to +200 bp) construct in NA‐ (Figure 3B, *P* ≤ .05) and NA‐ASM (Figure 3D; *P* ≤ .05) compared to the promoter‐less and (−100 to +200 bp) constructs. This activity was equivalent in A‐ and NA‐ASM and consistent with basal CTGF expression. The activity of the longer CTGF promoter constructs did not differ when compared to the −400 bp construct in either NA‐ (Figure 3B) or A‐ASM cells (Figure 3D) suggesting basal expression was regulated proximal to the transcriptional start site. There was no enhancement of the CTGF promoter activity after TGF‐β treatment in either NA‐ (Figure 3B) or A‐ ASM cells (Figure 3D) nor did a new regulatory element emerge in the full‐length or any of the truncated mutants. This was in direct contrast to CTGF mRNA expression which increased in both NA (Figure 3C)‐ and A (Figure 3E)‐ASM cells after TGF‐β treatment in the presence of all transfections.

### 3.3 Tissue specific genetic elements indicate CTGF regulation in lung tissue is unique

To assess whether our promoter construct was indeed inducible by TGF‐β and to determine whether CTGF regulation in ASM was different to other tissues, we transfected NIH‐3T3 cells and examined luciferase activity. Previous reports have shown that CTGF promoter‐luciferase reporter constructs increase activity 2‐ to 4‐fold when NIH‐3T3 cells are stimulated with TGF‐β.^25^, ^27^, ^33^, ^34^ Indeed, treatment of transfected NIH‐3T3 fibroblasts with TGF‐β induced a 2‐fold increase in luciferase expression compared to unstimulated cells (Figure S1) indicating our CTGF promoter construct (−400 to +200) was indeed inducible but just not in human ASM cells.

**Figure 4:**
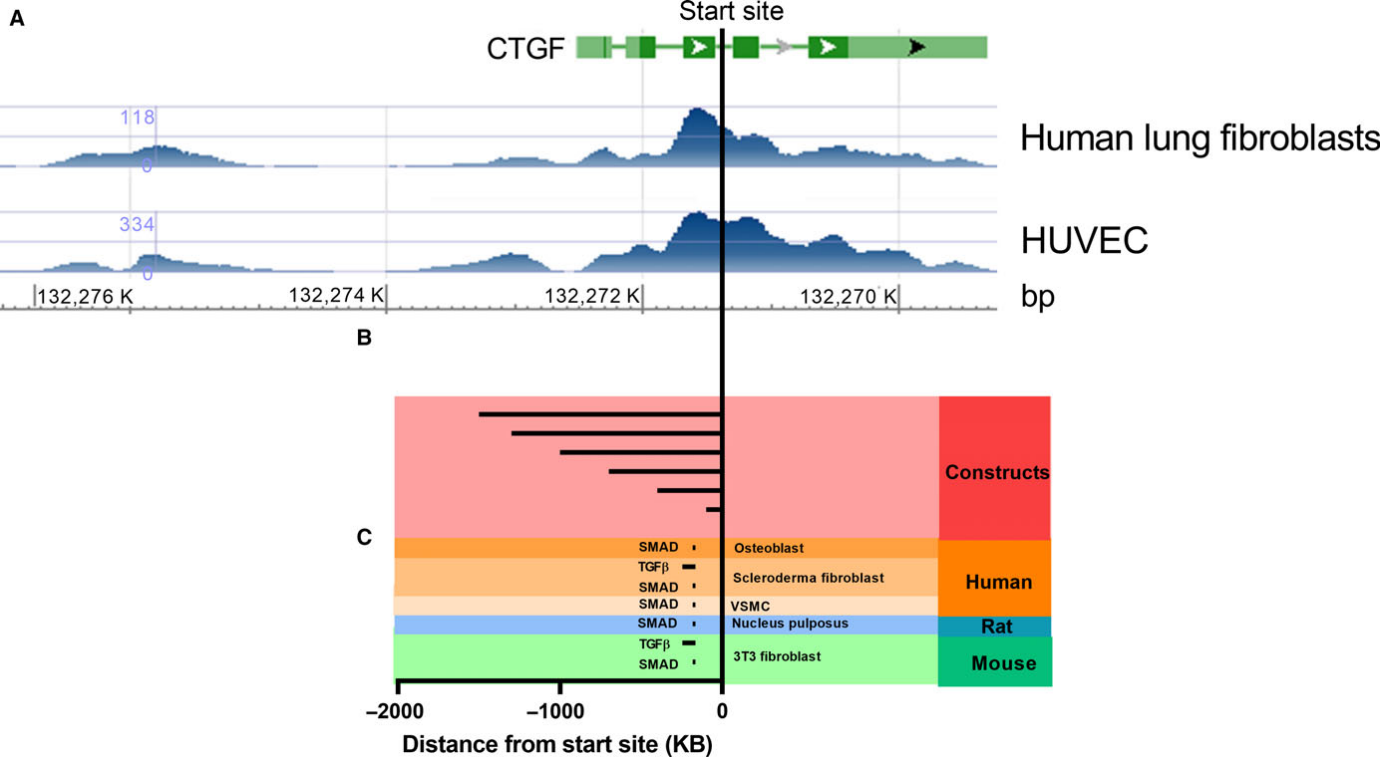
Differential usage of promoter elements endows cell‐type specific regulation of connective tissue growth factor (CTGF) in asthmatic airway smooth muscle. A, H3K27ac profiling from human lung fibroblasts and HUVECs surrounding the CTGF gene by ChIP‐Seq. B, Plasmid CTGF promotor construct used in this project. C, Validated SMAD and transforming growth factor (TGF)‐β transcription factor binding sites across difference species. HUVECs, human umbilical vein endothelial cells; SMAD, similar to mothers against decapentaplegic. Analysis was performed using the ENCODE database.

Having found that the CTGF promoter regulation in human ASM cells differed from that reported in other cell lines,^25^, ^27^, ^33^, ^34^ we investigated regions of transcription factor binding activity surrounding the CTGF transcriptional start site to look for additional regulatory elements. This analysis was conducted by investigating H3K27Ac binding (a marker of transcription factor binding). For this analysis, we used human lung fibroblasts as previously no differences in gene expression were detected between lung fibroblasts and ASM cells, indicating highly similar gene expression regulation.^35^ There was a strong region of activity immediately upstream of the CTGF transcriptional start site in HUVECs (−1300 to −200 bp), which was less active in human lung fibroblasts (Figure 4A). This region spanned the −1500 bp promoter construct we had analysed (Figure 4B), and contained several validated SMAD and TGF‐β response elements previously reported to drive CTGF expression in other species (Figure 4C, Table 3).^25^, ^27^, ^36^ However, an alternate genomic region 5′ to the (−1300 to −200 bp) site (−4200 to −2400 bp) showed robust H3K27Ac binding in lung cells but relatively low activity in HUVECs (Figure 4A). These findings suggest that this region may be responsible for the alternative regulation of CTGF expression in human lung cells.

**Table 3:** TGF‐β transcription factor binding sites across difference species

Cells type	Transcription factor	Binding site in CTGF promoter
NIH‐3T3 fibroblast	Smad^33^, ^34^, ^58^	−173 to −166
	TGF‐β responsive element^34^, ^58^	−246 to −166
	Ets1^59^	−126 to −77
Nucleus pulposus cells	Smad^26^	−173 to −166
C3H10T1/2	Smad^25^	−173 to −166
Scleroderma fibroblast	Smad^58^	−173 to −166
	TGF‐β responsive element^58^	−246 to −166
Osteoblast	Smad^60^	−173 to −166
	Ets1^42^	−126 to −77

CTGF, connective tissue growth factor; TGF, transforming growth factor.

### 3.4 CTGF mRNA stability is enhanced in asthmatic ASM cells

**Figure 5:**
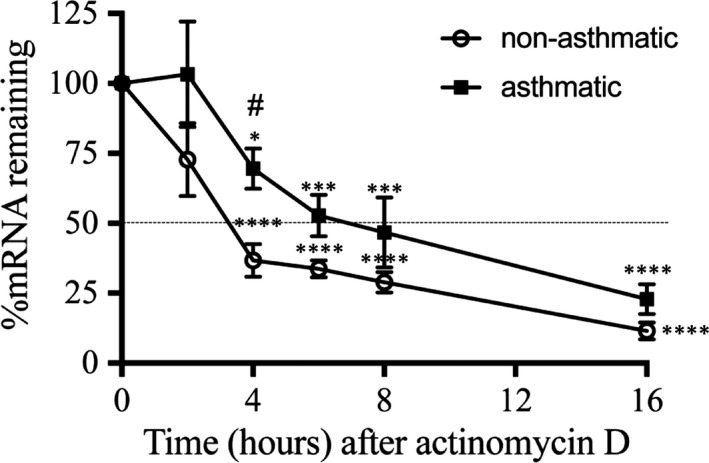
Connective tissue growth factor (CTGF) mRNA stability is enhanced in A‐ASM cells. NA‐ (n = 4) and A‐ASM cells (n = 5) were treated with TGF‐β (1 ng/mL) with actinomycin D (10 μg/mL) added after 8 h for up to 16 h. CTGF mRNA expression was measured by Q‐PCR to assess the rate of turnover. *Means significant difference in CTGF mRNA expression to time 0, *P < .05, ***P < .001, ****P < .0001. #*P* < .05 indicates a significant difference between NA‐ and A‐ASM. ASM, asthmatic airway smooth muscle; TGF, transforming growth factor

Ibrul Chowdhury et al previously reported that FBS‐induced CTGF mRNA expression was regulated by both new transcription and mRNA stabilization in primary bladder smooth muscle cells.^37^ To further characterize the regulation of CTGF in A‐ and NA‐ASM cells, we compared the kinetics of CTGF RNA turnover induced by TGF‐β. In NA‐ASM cells, CTGF transcripts were rapidly degraded, with a half‐life of 3 hours (Figure 5). In contrast, the half‐life of CTGF mRNA in A‐ASM cells was prolonged (7 hours) suggesting that the mRNA stability was enhanced in the A‐ASM cells. Linear regression showed that the degradation rate after 6 hours between A‐ (*k* = −2.99) and NA‐ (*k* = −2.42) ASM cells was similar. However, in NA‐ASM cells, the degradation of CTGF mRNA in the first 6 hours was 20.3% higher than in the A‐ASM cells (*k* = −15.82 vs *k* = −12.6, respectively) suggesting enhanced stability and slower degradation are the source of the longer mRNA half‐life in A‐ASM cells. These data suggested that TGF‐β‐induced CTGF mRNA in A‐ASM cells was more stable than NA‐ASM cells providing a possible explanation for the differential expression of TGF‐β‐induced CTGF between A‐ and NA‐ASM cells.

### 3.5 CTGF gene expression is unchanged in mild asthmatic patients but relates to BM thickness

**Figure 6:**
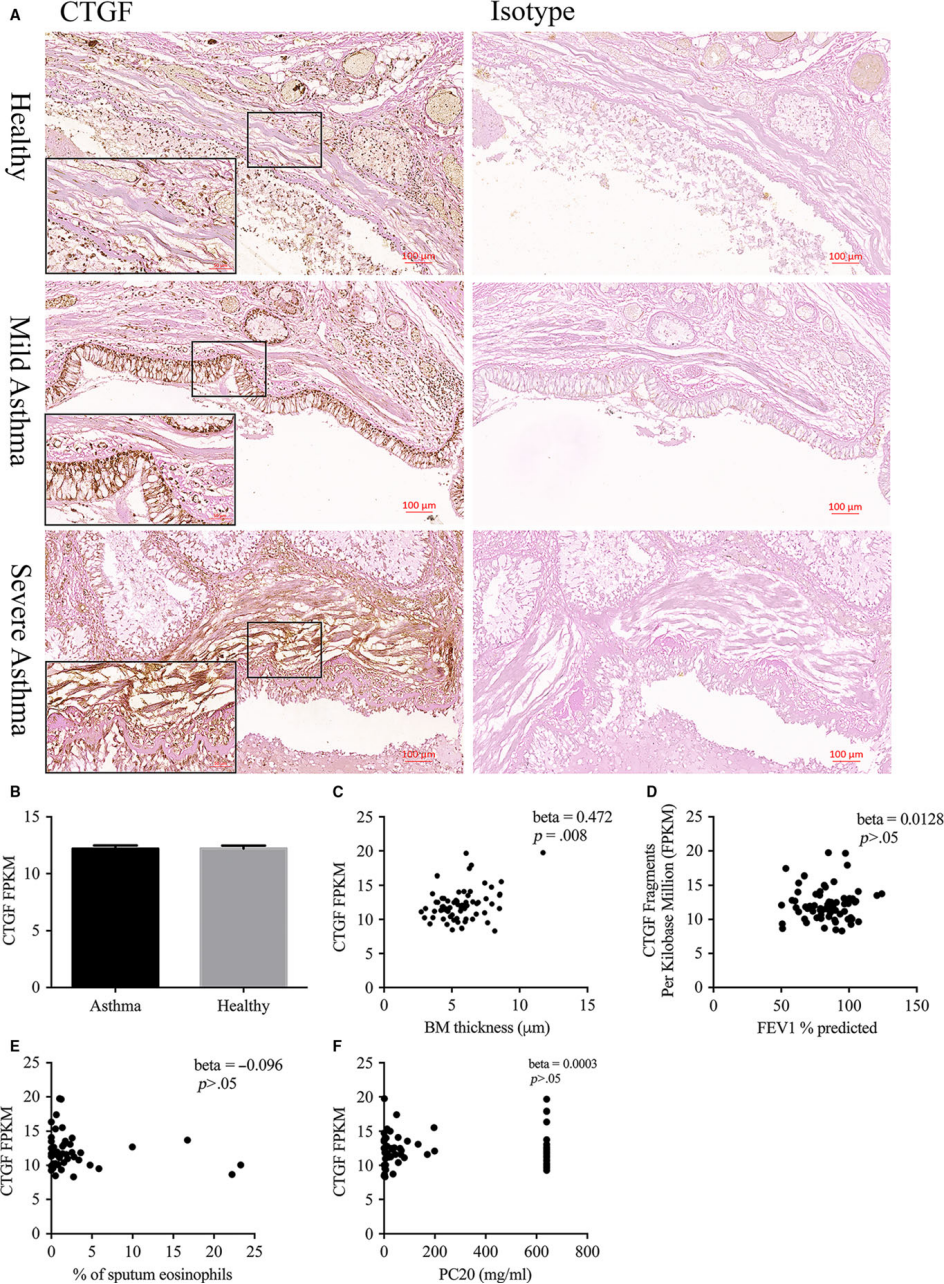
Connective tissue growth factor (CTGF) expression and correlations with clinical indices in asthmatic patients. A, CTGF expression was assessed by immunohistochemistry in human lung tissue (n = 5 for healthy control, mild asthma and severe asthma). Representative images shown for each group. B‐E, CTGF mRNA expression fragments per kilobase million (FPKM) was detected in bronchial biopsies from healthy controls and mild asthmatic patients (B). A linear model comparing the association between CTGF expression in asthmatic bronchial biopsies and BM thickness (μmol/L) (C), FEV1% predicted (D), % of sputum eosinophils (E) and PC20 mg/mL (F) was conducted correcting for age, gender and smoking status. β, correlation co‐efficient; *P*, significance value of the correlation. BM basement membrane, FEV1% predicted forced expiratory volume in 1 s percentage predicted, PC20 the concentration of methacholine needed to produce a 20% fall in FEV(1) from baseline. See Ref. 32 (Table 1) for lung function on this cohort

Immunohistochemical staining showed CTGF protein expression was concentrated in the ASM area in human lung tissue, with enhanced detection visible in asthmatic tissues, particularly from severe asthma patients (Figure 6A). No difference in CTGF mRNA expression (Figure 6B) was detected between bronchial biopsies derived from mild to moderately severe asthma patients (n = 69) and healthy controls (n = 77). In addition, within asthmatic patients, we found a significant relationship between CTGF expression and BM thickness (β ± SE 0.472 ± 0.174, *P* = .008, Figure 6C) suggesting ASM‐derived CTGF expression may influence airway narrowing and remodelling in asthma. In contrast, higher CTGF expression in asthmatic patients was not associated with lower FEV1% predicted (Figure 6D), more severe bronchial hyper‐responsiveness (Figure 6F) or higher % eosinophil levels in sputum (Figure 6E).

## 4 DISCUSSION

This is the first study that has focused on the molecular regulation of CTGF mRNA in primary ASM cells. Tissue localization in asthmatic airways indicated that the ASM cells were the primary location of CTGF expression. We have shown that, unlike other cell types, regulation of CTGF mRNA expression in primary ASM cells may not be located in the first 1500 bases in the CTGF promoter, but rather lie some distance upstream. In addition, post‐transcriptional regulation of mRNA stability plays a role in the differential TGF‐β‐induced CTGF mRNA expression in A‐ and NA‐ASM cells. CTGF expression in asthmatic patients correlated with the degree of BM thickening, suggesting CTGF may contribute to the mechanisms driving airway remodelling.

In our study, we observed greater CTGF expression in airway tissues taken from lung segments of sheep chronically exposed to HDM allergen. These findings were consistent with other in vivo studies that have reported that CTGF mRNA and protein are up‐regulated in animal models of allergic airways disease.^38^, ^39^ CTGF was also enhanced in lung tissue from severe asthmatic patients, compared to non‐asthmatic controls. The ASM cells, as the primary source of CTGF in lung tissue, are uniquely positioned to drive remodelling (new blood vessel formation and BM thickening) as they lie immediately below the lamina propria, a major location of vascular expansion and ECM deposition in remodelling airways.

To illuminate the cause of the difference in regulation of CTGF mRNA between A‐ and NA‐ASM cells, we investigated transcriptional and post‐transcriptional regulation of CTGF in primary ASM cells. The well‐characterized TGF‐β responsive elements in the CTGF promoter^25^, ^27^, ^33^, ^34^ appeared to not be involved in the regulation of CTGF in ASM cells. However, the CTGF promoter constructs used in this study do not integrate into the chromatin. As such, the promoter constructs are not subject to the same epigenetic controls as the TGF‐β responsive elements in the endogenous gene which may account for some of the discrepancy. Our discovery of a novel CTGF transcriptional regulatory region, upstream of what is considered to be the core promoter region, in lung fibroblasts opens new realms in the tissue specific regulation of CTGF expression. Given the similarities in gene expression, we speculate that this mechanism is likely to play a role in the unique regulation of CTGF in lung mesenchymal cells; however, the absence of ASM data precluded our validation in ASM directly and this is a limitation of our study. We have previously shown TGF‐β induced CTGF through activation of the extracellular signal‐regulated kinase (ERK) and phosphatidylinositol 3‐kinase (PI3K) signalling pathways in ASM cells. ERK is known to be linked to SMAD2/3 activation^36^ and likely targets the traditional core promoter region (−1300 to −200 bp) of CTGF which appears to be SMAD sensitive (Figure 4). However, PI3K has not been associated with SMAD 2/3 signalling to date and therefore may target the alternative promoter regulatory region we have identified in this study. Moreover, how these distant genomic elements are recruited to the core promoter to modulate CTGF expression is unknown and may be influenced by epigenetic modification of histones (particularly K^27^ acetylation) which is readily acknowledged to be different in asthmatic and healthy airways.^40^, ^41^ Further research is necessary to identify the transcriptional regulatory elements, potentially within the −4200‐ to −2400‐bp region, activated by these alternative signalling pathways for driving CTGF expression.

The similarities in basal promoter activity in A‐ and NA‐ASM were just as surprising as the lack of TGF‐β responsiveness in the CTGF promoter. We identified that the basal promoter in ASM is located between −100 and −400 bp. This region contains predicted binding sites for SMAD, AP1, TGF‐β element, Ets1, NF‐1‐like sequence, TIE‐like site amongst others. Some of these factors have previously been implicated in TGF‐β signalling^26^, ^27^, ^42^ whilst others have not. The similarity of basal CTGF promoter activity in A‐ and NA‐ASM underscores the importance of the inflammatory milieu in the asthmatic airway in directing the phenotype of ASM during disease.

We also showed that CTGF mRNA stability was enhanced in A‐ASM cells. Chowdhury and colleagues previously showed this to be mediated by p38 in bladder smooth muscle cells.^37^ However, our previous data conclusively showed this pathway is not involved with CTGF regulation by TGF‐β in A‐ASM.^22^ The mechanism underlying this enhanced CTGF mRNA stability is currently unknown.

The dysregulation of CTGF in asthmatic airways may have profound consequences for disease progression, as suggested by the association of CTGF gene expression levels with BM thickening in our patient cohort. One limitation in our study is that the biopsies from which we obtained the gene signal were of a mixed cell population and we have no information about the ASM content in each biopsy. This may have altered the CTGF gene signal as CTGF is also expressed by airway epithelial cells and fibroblasts^43^, ^44^, ^45^, ^46^, ^47^ and it is not known if these levels are also altered in asthma. If the epithelial gene expression of CTGF is not increased in asthma, this may have reduced the strength of the association we observed with BM thickness. The BM is 2‐ to 3‐fold thicker in asthmatic compared to healthy airways and is associated with increased airway resistance, limitations to airflow and decreased lung function.^48^, ^49^ Association of BM thickening with poor clinical outcome is somewhat controversial with adults^32^, ^50^ but shows better correlation in children.^50^, ^51^, ^52^ The thicker BM of asthmatic airways also has an altered elastic modulus compared to healthy airways.^53^ The increased ECM stiffness that accompanies such a change is likely to contribute to the pro‐remodelling environment found in asthmatic airways as stiffer matrices promote angiogenesis^54^, ^55^ and ASM cell proliferation.^53^ Indeed, stiffer matrices may also enhance CTGF expression through Taz activation,^56^, ^57^ completing a positive feedback loop in the asthmatic airway that would co‐ordinate all aspects of airway remodelling (ASM bulk, neovascularization and BM thickening).

In conclusion, our data strongly suggest that the unique regulatory mechanisms that underpin the enhanced CTGF expression in A‐ASM are pivotal for the development of airway remodelling. Thus, CTGF represents an underappreciated target for future therapeutic intervention addressing an aspect of disease pathogenesis currently not effectively treated by existing approaches.

## CONFLICTS OF INTERESTS

The authors confirm there are no conflict of interests in this study.
