# Supplementary figures and images for: Unique mechanisms of connective tissue growth factor regulation in airway smooth muscle in asthma: Relationship with airway remodelling
Source: J Cell Mol Med. 2018 Mar 7;22(5):2826–37. doi: 10.1111/jcmm.13576 (PMC5908101; doi:10.1111/jcmm.13576)

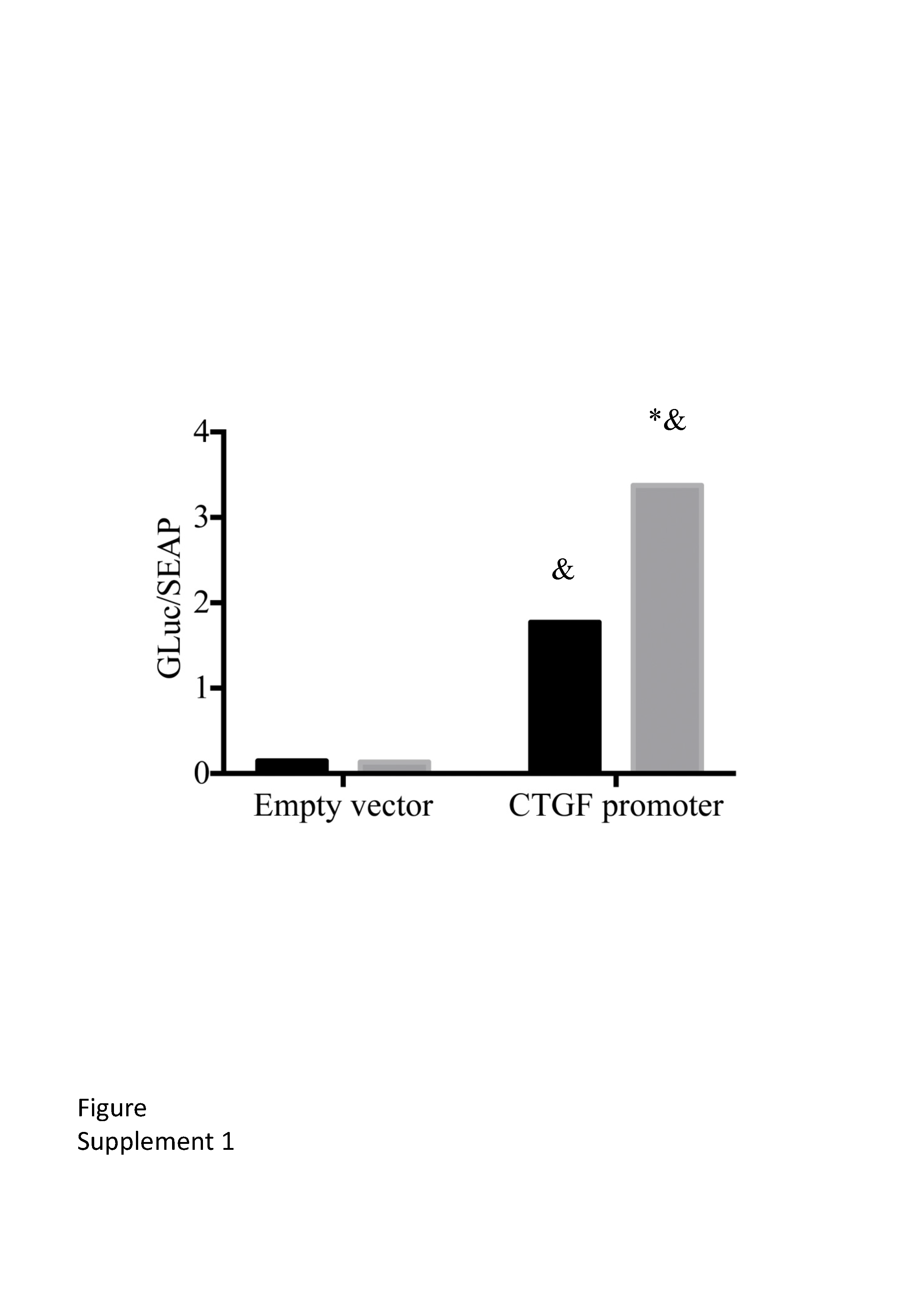

Supplement: Supplementary file 1 [file JCMM-22-2826-s001.tiff]
