# Supplementary material for: Unique mechanisms of connective tissue growth factor regulation in airway smooth muscle in asthma: Relationship with airway remodelling
Source: J Cell Mol Med. 2018 Mar 7;22(5):2826–37. doi: 10.1111/jcmm.13576 (PMC5908101; doi:10.1111/jcmm.13576)
Supplement: Supplementary file 2 [file JCMM-22-2826-s002.docx]

**SUPPLEMENTAL INFORMATION**

**MATERIALS AND METHODS**

Patients

We obtained high-quality RNAseq data from 184 biopsies. Biopsies were derived from 77 healthy individuals and 107 current or former asthma patients. All patients originated from cohorts investigated earlier by our research group, and a set of previously acquired clinical and genotype data is available (Broekema et al. 2010, 2011, Vonk et al. submitted).

All 107 patients had a previous doctors’ diagnosis of asthma, documented reversibility and AHR to histamine (PC20 =< 32 mg/mL). We split the persistent asthma patients on the basis of use of inhaled corticosteroids (ICS), as corticosteroids are known to have strong impact on cell physiology and thus potentially act as a confounder in our analysis (Dorscheid et al. 2006, Wadsworth et al. 2006, Liu et al. 2013,). We considered patients to be in clinical remission if they had not had an asthma attack or wheeze in the last 3 years, and did not use asthma medication (ß-agonists and ICS) for the past 3 months. If individuals, in addition to the previous criteria did not have AHR to histamine and AMP (> 32 mg/mL in 30 s tidal breathing and > 320 mg/mL in 2 min tidal breathing, respectively) and had no signs of airflow obstruction (FEV1%predicted > 80% pre-bronchodilator or >90% post-bronchodilator), they were considered to be in complete remission.

The control cohort of 77 respiratory healthy individuals was derived from the NORM study (Boudewijn et al. 2015). In this study, current smokers and never smokers older than 18 years were recruited. Patients were considered respiratory healthy if they had no respiratory symptoms, no history of respiratory disease and normal pulmonary function. Normal pulmonary function was defined as a post-bronchodilator FEV1/FVC higher than lower limit of normal, absence of AHR to methacholine (PC20 <16mg/mL) and absence of reversibility (FEV1%predicted to salbutamol < 10%). Patients were excluded if they used inhaled or oral corticosteroids within the last 5 years, or during 5 years of their lives.

The study protocol was approved by the local medical ethics committee. All patients gave their written informed consent.

RNA extraction, Sample preparation and High-throughput sequencing

Bronchial biopsies were taken from segmental divisions of the main bronchi. Biopsies frozen in Tissuetek at -80°C were thawed at room temperature and cut from the blocks when they were semi-solid. Total RNA was extracted using AllPrep DNA/RNA Mini kit (Qiagen). Samples were lysed in 600 μl RLT-plus buffer using an IKA Ultra Turrax T10 Homogenizer, and RNA was purified according to the manufacturer’s instructions. RNA samples were dissolved in 30 μl RNase-free water. Concentrations and quality of RNA were checked using a NanoDrop-1000 and run on a Labchip GX (Perkin Elmer).

RNA samples were further processed using the TruSeq Stranded Total RNA Sample Preparation Kit (Illumina), using an automated procedure in a Caliper Sciclone NGS Workstation (Perkin Elmer). In this procedure, all cytoplasmic and mitochondria rRNA was removed (RiboZero Gold kit). The obtained cDNA fragment libraries were loaded in pools of multiple samples unto an Illumina HiSeq2500 sequencer using default parameters for paired-end sequencing (2 × 100 bp).

Gene expression quantification

The trimmed fastQ files where aligned to build b37 of the human reference genome using HISAT (version 0.1.5) allowing for 2 mismatches (Kim et al. 2015). Before gene quantification SAMtools (version 1.2) was used to sort the aligned reads (Li et al. 2009). The gene level quantification was performed by HTSeq (version 0.6.1p1) using –mode=union –stranded=no and using Ensembl version 75 as gene annotation database (Anders et al. 2015).
